# Supplementary material for: Chinese CO2 emission flows have reversed since the global financial crisis
Source: Nat Commun. 2017 Nov 23;8:1712. doi: 10.1038/s41467-017-01820-w (PMC5700086; doi:10.1038/s41467-017-01820-w)
Supplement: Supplementary file 1 — Description of Additional Supplementary Files [file 41467_2017_1820_MOESM1_ESM.docx]

**Description of Additional Supplementary Files**

File Name: Supplementary Data 1

Description: Definition of world regions.

File Name: Supplementary Data 2

Description: Concordance of sectors for provincial IOTs and Chinese MRIO.

File Name: Supplementary Data 3

Description: Concordance of sectors for Chinese MRIO and GTAP database.

File Name: Supplementary Data 4

Description: CO2 emission inventories for China’s 30 provinces in 2007.

File Name: Supplementary Data 5

Description: CO2 emission inventories for China’s 30 provinces in 2010.

File Name: Supplementary Data 6

Description: CO2 emission inventories for China’s 30 provinces in 2012.

File Name: Supplementary Data 7

Description: China's 2012 MRIO for 30 provinces and 30 sectors.
