# Supplementary material for: Chinese CO2 emission flows have reversed since the global financial crisis
Source: Nat Commun. 2017 Nov 23;8:1712. doi: 10.1038/s41467-017-01820-w (PMC5700086; doi:10.1038/s41467-017-01820-w)
Supplement: Supplementary file 2 — Supplementary Information [file 41467_2017_1820_MOESM2_ESM.pdf]

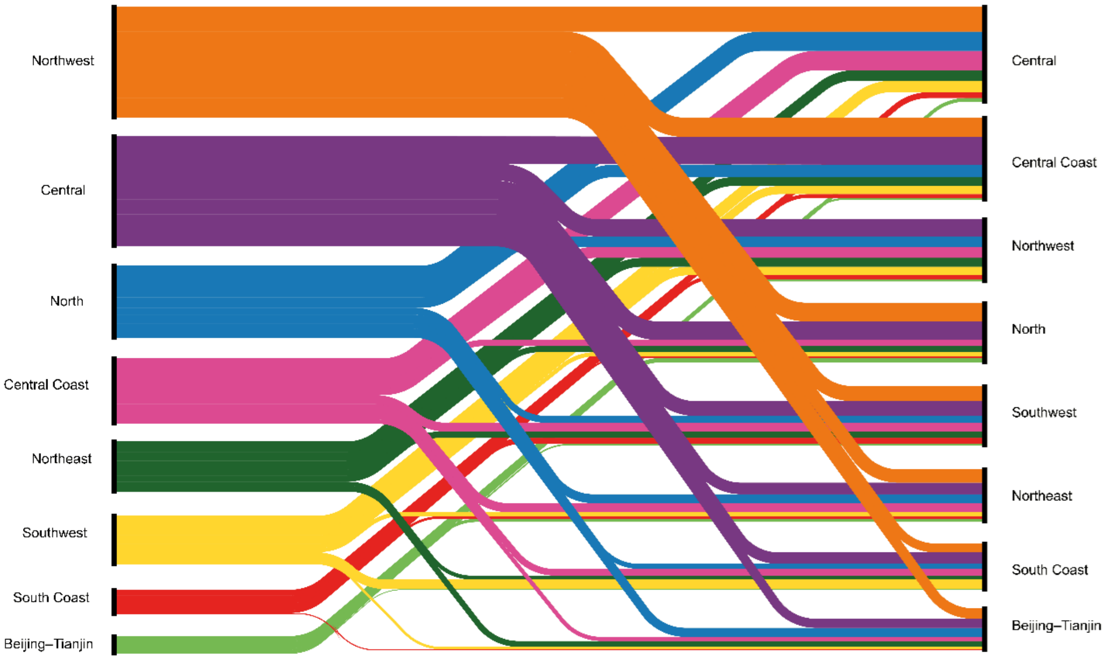

Supplementary Figure 1 Interregional carbon emission flows within China in 2012. Northwest had the largest emission outflows, while Central and Central Coast had the largest emission inflows. Large CO_2 emissions related to goods and services consumed in highly developed eastern coastal provinces are imported from less developed provinces in central and western China.

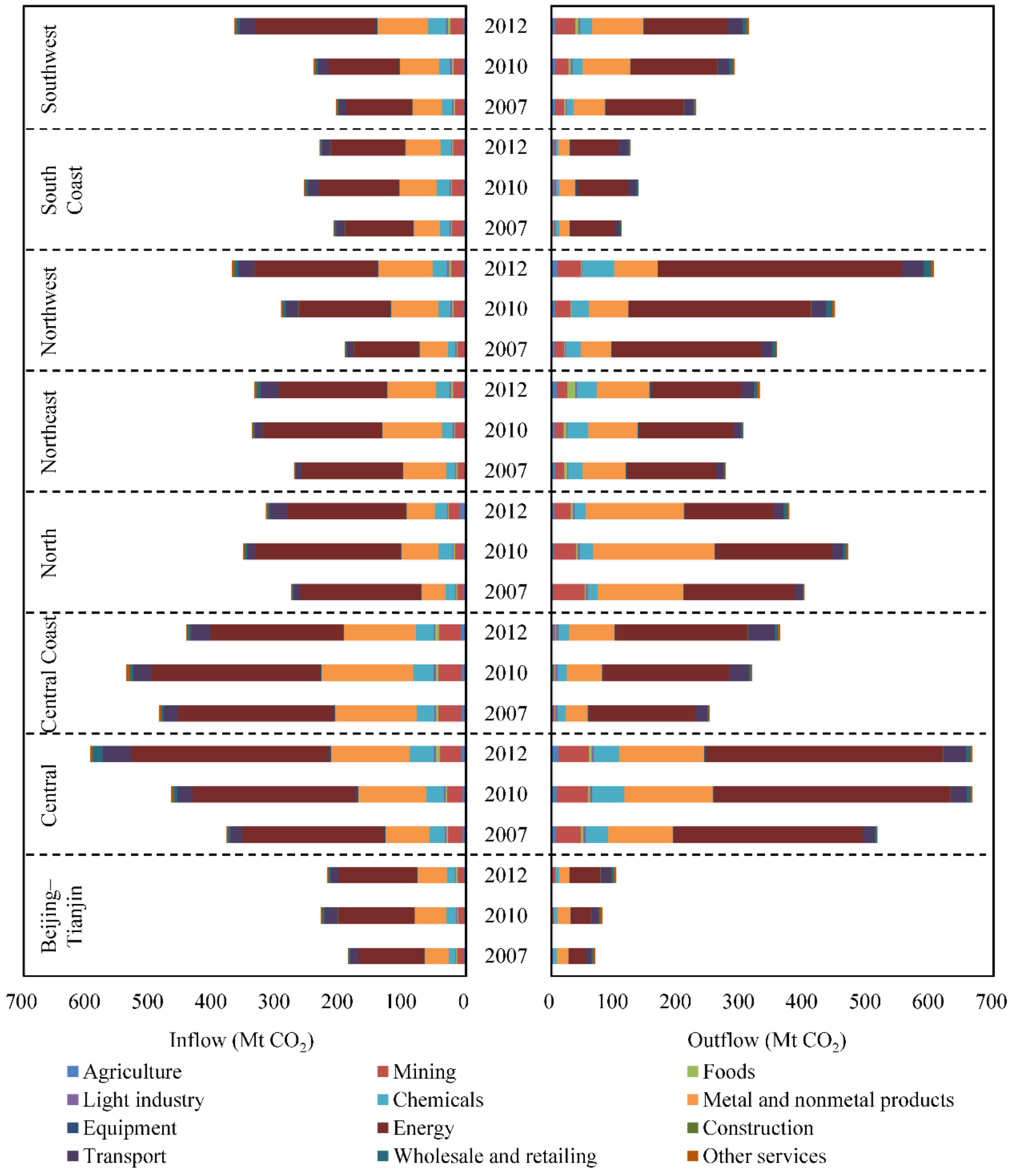

Supplementary Figure 2 Emission outflows and inflows of eight Chinese regions in 2007, 2010 and 2012.

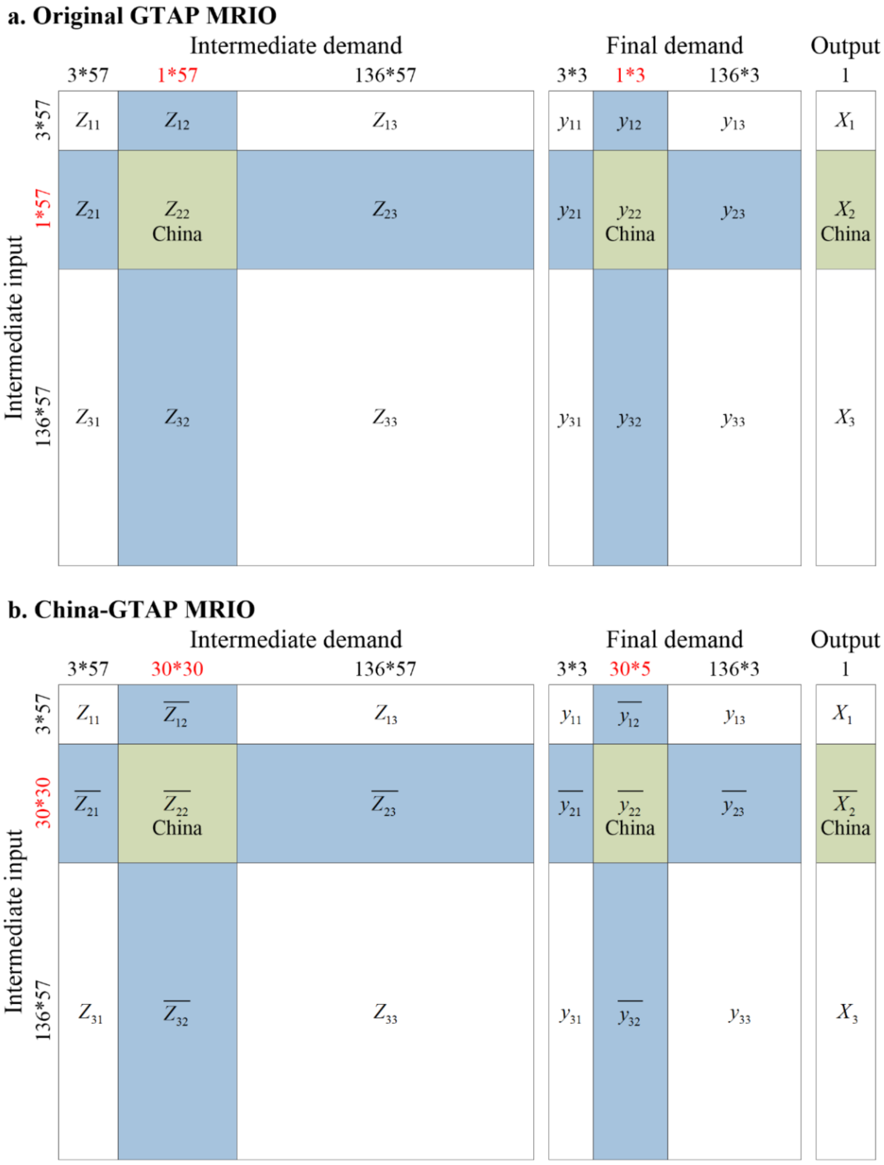

Supplementary Figure 3 The structure of original GTAP MRIO and China-MRIO. The figures demonstrate the dimensions for the matrices. Matrices in green are China's domestic structure, matrices in blue are China's imports and exports, and matrices in white do not change. In this study, we build a new China-MRIO by combing Chinese MRIO for 30 provinces and 30 sectors with GTAP MRIO for 140 countries and 57 sectors. First of all, matrices for China's domestic structure in GTAP are replaced by Chinese MRIO directly (in green). Z_{22} is China's domestic production structure, y_{22} is China's domestic final demand, and X_2 is China's output. Second, matrices for China's international exports and imports are calculated under the assumption that international exports (or imports) of a sector in a province are distributed among all foreign countries in the same proportion as China's exports (or imports) of the sector (in blue). Z_{12} and Z_{32} are China's imports for intermediate use, Z_{21} and Z_{23} are China's exports for intermediate use, y_{12} and y_{32} are China's imports for final use, and y_{21} and y_{23} are China's exports for final use. Third, matrices for other countries do not change (in white).

Supplementary Table 1 Territory- and consumption-based CO_2 emissions for Chinese 30 provinces (Unit: Mt CO_2).

No.	Provinces	Territory-based emissions			Consumption-based emissions		
		2007	2010	2012	2007	2010	2012
1	Beijing	88	86	80	159	171	159
2	Tianjin	98	129	149	108	177	185
3	Hebei	482	584	648	246	389	451
4	Shanxi	324	378	431	167	224	289
5	Inner Mongolia	321	441	578	112	243	301
6	Liaoning	341	421	430	213	325	350
7	Jilin	157	186	214	186	242	232
8	Heilongjiang	174	192	241	160	195	258
9	Shanghai	165	177	182	216	248	177
10	Jiangsu	426	522	592	319	432	481
11	Zhejiang	285	312	329	327	342	323
12	Anhui	175	231	279	151	191	203
13	Fujian	143	176	205	122	170	190
14	Jiangxi	108	125	137	154	148	144
15	Shandong	602	698	767	472	600	709
16	Henan	381	454	452	244	357	437
17	Hubei	221	283	322	179	218	328
18	Hunan	198	220	237	166	204	261
19	Guangdong	360	414	440	331	421	466
20	Guangxi	111	144	169	96	148	188
21	Hainan	19	24	31	18	22	40
22	Chongqing	86	122	139	97	118	172
23	Sichuan	175	245	266	176	207	274
24	Guizhou	152	170	197	83	105	140
25	Yunnan	144	170	179	99	147	188
26	Shaanxi	130	190	226	113	191	234
27	Gansu	87	111	134	64	85	107
28	Qinghai	22	27	38	26	30	41
29	Ningxia	62	90	129	43	70	72
30	Xinjiang	116	155	233	99	141	196
	**Total**	**6154**	**7476**	**8454**	**4947**	**6565**	**7600**

Supplementary Table 2 Sector classification of pricing data for global MRIO tables.

No.	Abbreviations	Sectors
1	ISIC A-B	Agriculture, hunting, forestry, fishing
2	ISIC C-E	Mining, Manufacturing, Utilities
3	ISIC D	Manufacturing
4	ISIC F	Construction
5	ISIC G-H	Wholesale, retail trade, restaurants and hotels
6	ISIC I	Transport, storage and communication
7	ISIC J-P	Other Activities

Note: We deflated all the tables to 2012 prices using the double deflation method[1]. The pricing data for China's IOTs were gotten from the China Statistics Yearbook[2, 3, 4], while the pricing data for China's imports and global MRIO tables were obtained from the National Account Main Aggregates Database[5]. The National Account Main Aggregates Database provides pricing data for seven sectors, which are adapted to 57 sectors of global MRIO. In addition, the import value of China was deflated by the weighted average price deflators of all regions (excluding China). The weight for one region was in proportion to China's imports from the region. It needs to be noted that there are several drawbacks related to double deflation method, although this method is widely accepted. Firstly, by adopting this method, most sectors are assumed to produce one homogeneous product, each sector's gross output and intermediate and final demand are deflated by this sector's price index[6]. However, most sectors consist of more than one good, therefore to use the price index of certain goods to represent the entire sector is not always appropriate[7]. Secondly, value-added is obtained as the difference between the total input and intermediate input in each sector. Consequently, it is not accurate to use value-added to balance the input-output table after the deflation[8].

Supplementary Table 3 Emission factors for different energy types for calculating CO_2 emissions.

No.	Energy types	Net calorific value (PJ / $10^4\ t, 10^8\ m^3$)	Carbon content (Mt CO_2 / PJ)	Oxygenation efficiency (%)
1	Raw coal	0.20908	0.087464	88.535
2	Cleaned coal	0.26344	0.087464	88.535
3	Other washed coal	0.15393	0.087464	88.535
4	Briquettes	0.17796	0.087464	88.535
5	Coke	0.28435	0.104292	97.000
6	Coke oven gas	1.63080	0.071414	99.000
7	Other gas	0.84290	0.071414	99.000
8	Other coking products	0.28435	0.091212	97.000
9	Crude oil	0.41816	0.073284	98.000
10	Gasoline	0.43124	0.069253	98.000
11	Kerosene	0.43124	0.071818	98.000
12	Diesel oil	0.42652	0.074017	98.000
13	Fuel oil	0.41816	0.077314	98.000
14	Liquefied petroleum gas (LPG)	0.50179	0.063024	99.000
15	Refinery gas	0.46055	0.073284	99.000
16	Other petroleum products	0.41816	0.074017	98.000
17	Nature gas	3.89310	0.056062	99.000
18	Non-fossil Heat	0.01000	0.000000	0.0000
19	Non-fossil Electricity	0.36000	0.000000	0.0000
20	Other energy	0.29308	0.000000	0.0000

Note: The Intergovernmental Panel on Climate Change (IPCC) reference approach is used to calculate the CO_2 emissions from energy combustion. The emission factors (i.e. net calorific value, carbon content, and oxygenation efficiency) pay a critical role in calculations. Researchers usually use the IPCC default value[9]. However, this default value may overestimate Chinese CO_2 emissions[10, 11]. In this study, we use the emission factors from our previous research[10]. The factors are measured based on 602 coal samples from the 100 largest coal-mining areas in China and are assumed to be more accurate than the IPCC default value.

Supplementary References:

1. UNSD. Handbook of input-output table compilation and analysis. http://unstats.un.org/unsd/publication/SeriesF/SeriesF_74E.pdf (1999).
2. National Bureau of Statistics. *China Statistical Yearbook 2007*. (China Statistics Press, Beijing, 2007).
3. National Bureau of Statistics. *China Statistical Yearbook 2011*. (China Statistics Press, Beijing, 2011).
4. National Bureau of Statistics. *China Statistical Yearbook 2015*. (China Statistics Press, Beijing, 2015).
5. UNSD. National accounts main aggregates database. http://unstats.un.org/unsd/snaama/Introduction.asp (2016).
6. Dietzenbacher, E. & Hoen, A. R. Deflation of input-output tables from the user's point of view: a heuristic approach. *Rev. Income Wealth* **44**, 111-122 (1998).
7. Sevaldson, P. Price changes as causes of variations in input-output coefficients. In: *Advances in Input-Output Analysis* (eds. Polenske, K. R., Skolka, J. V.). (Ballinger, Cambridge, MA, 1976).
8. Wolff, E. N. Productivity measurement within an input-output framework. *Reg. Sci. Urban Econ.* **24**, 75-92 (1994).
9. IPCC. 2006 IPCC guidelines for national greenhouse gas inventories. http://www.ipcc-nggip.iges.or.jp/public/2006gl/index.html (2006).
10. Liu, Z., *et al.* Reduced carbon emission estimates from fossil fuel combustion and cement production in China. *Nature* **524**, 335-338 (2015).
11. Mi, Z., *et al.* Pattern changes in determinants of Chinese emissions. *Environ. Res. Lett.* **12**, 074003 (2017).
